# Prediction of outcomes after cardiac arrest by a generative artificial intelligence model

**DOI:** 10.1016/j.resplu.2024.100587

**Published:** 2024-02-22

**Authors:** Simon A. Amacher, Armon Arpagaus, Christian Sahmer, Christoph Becker, Sebastian Gross, Tabita Urben, Kai Tisljar, Raoul Sutter, Stephan Marsch, Sabina Hunziker

**Affiliations:** a Intensive Care Medicine, Department of Acute Medical Care, University Hospital Basel, Basel, Switzerland; b Medical Communication and Psychosomatic Medicine, University Hospital Basel, Basel, Switzerland; c Emergency Medicine, Department of Acute Medical Care, University Hospital Basel, Basel, Switzerland; d Medical Faculty, University of Basel, Basel, Switzerland; e Division of Neurophysiology, Department of Neurology, University Hospital Basel, Basel, Switzerland; f Post-Intensive Care Clinic, University Hospital Basel, Basel, Switzerland

**Keywords:** Artificial intelligence, Cardiac arrest, Cardiopulmonary resuscitation, Mortality prediction, Neurological outcome

## Abstract

**Aims:**

To investigate the prognostic accuracy of a non-medical generative artificial intelligence model (Chat Generative Pre-Trained Transformer 4 - ChatGPT-4) as a novel aspect in predicting death and poor neurological outcome at hospital discharge based on real-life data from cardiac arrest patients.

**Methods:**

This prospective cohort study investigates the prognostic performance of ChatGPT-4 to predict outcomes at hospital discharge of adult cardiac arrest patients admitted to intensive care at a large Swiss tertiary academic medical center (COMMUNICATE/PROPHETIC cohort study). We prompted ChatGPT-4 with sixteen prognostic parameters derived from established post-cardiac arrest scores for each patient. We compared the prognostic performance of ChatGPT-4 regarding the area under the curve (AUC), sensitivity, specificity, positive and negative predictive values, and likelihood ratios of three cardiac arrest scores (Out-of-Hospital Cardiac Arrest [OHCA], Cardiac Arrest Hospital Prognosis [CAHP], and PROgnostication using LOGistic regression model for Unselected adult cardiac arrest patients in the Early stages [PROLOGUE score]) for in-hospital mortality and poor neurological outcome.

**Results:**

Mortality at hospital discharge was 43% (n = 309/713), 54% of patients (n = 387/713) had a poor neurological outcome. ChatGPT-4 showed good discrimination regarding in-hospital mortality with an AUC of 0.85, similar to the OHCA, CAHP, and PROLOGUE (AUCs of 0.82, 0.83, and 0.84, respectively) scores. For poor neurological outcome, ChatGPT-4 showed a similar prediction to the post-cardiac arrest scores (AUC 0.83).

**Conclusions:**

ChatGPT-4 showed a similar performance in predicting mortality and poor neurological outcome compared to validated post-cardiac arrest scores. However, more research is needed regarding illogical answers for potential incorporation of an LLM in the multimodal outcome prognostication after cardiac arrest.

## Introduction

In patients who survive sudden cardiac arrest until intensive care unit (ICU) admission, physicians are confronted with the challenging task of predicting neurological outcomes, as the presence and severity of hypoxic-ischemic brain injury are difficult to assess within the first days.^1^, ^2^, ^3^ Most deaths in cardiac arrest survivors occur due to the withdrawal of life-sustaining therapies (WLST) when a poor neurological outcome is assumed.^4^, ^5^ Hence, some cardiac arrest patients with a chance of substantial neurological recovery are at risk for premature WLST.^2^, ^4^, ^5^ Consequently, the present post-resuscitation care guidelines recommend a multimodal approach and delaying prognostication for at least 72 hours to decrease the risk of premature WLST.^6^ However, the multimodal approach does not integrate individual parameters (such as the time until the return of spontaneous circulation [ROSC] or lactate levels) as the predictive performance of individual parameters is limited.^7^ Therefore, it has been recommended to integrate several parameters into validated post-cardiac arrest scores, although these scores still have limited prognostic abilities for individual predictions of survival and/or neurological outcomes after cardiac arrest.^8^, ^9^, ^10^, ^11^ Artificial intelligence (AI) in its wider form might bring additional prognostic possibilities, as supervised machine learning algorithms in the form of artificial neural networks have shown promising prognostic performance in cardiac arrest patients.^12^, ^13^ Large generative artificial AI language models have recently gained worldwide attention with the release of Chat Generative Pre-trained Transformer 4 (ChatGPT-4),^14^ which is capable of deductive reasoning and writing complex texts about a wide range of topics.^15^, ^16^ Increasing evidence suggests that generative AI models like ChatGPT-4 might have the potential to answer complex medical problems.^16^, ^17^, ^18^, ^19^, ^20^, ^21^, ^22^, ^23^, ^24^ Unlike other large language models (LLM),^25^ the system was not developed for healthcare purposes. There are some recent studies using ChatGPT-4 as a medical decision aid in the acute care setting, for example, in the triage of patients in the emergency room.^26^, ^27^ However, the value of ChatGPT-4 for the prognostication of short-term outcomes in cardiac arrest patients remains unclear. To the best of our knowledge, there are currently no studies evaluating the value of LLMs for prognostication in patients after cardiac arrest. However, the potential of LLMs is promising, especially as LLMs might be provided with unstructured medical data.^28^ We thus compared the prognostic accuracy of the LLM ChatGPT-4 to predict mortality and neurological outcomes based on real-life data of a large cohort of adult cardiac arrest patients with three validated post-cardiac scores.^9^, ^10^, ^11^

## Methods

### Study setting & participants

At the University Hospital Basel, a Swiss tertiary teaching hospital and cardiac arrest center, adult in-hospital cardiac arrest (IHCA) and out-of-hospital cardiac arrest (OHCA) patients admitted to the ICU were consecutively included in an ongoing prospective cohort study to assess prognostication after cardiac arrest and long-term outcomes. The study procedures have been published previously in detail.^7^, ^29^, ^30^, ^31^, ^32^, ^33^, ^34^, ^35^, ^36^, ^37^, ^38^ All patients at the University Hospital Basel were treated in accordance with the corresponding guidelines of the European Resuscitation Council.^39^, ^40^, ^41^ The data analyzed in the present study was prospectively collected from October 2012 until December 2022. The data collection, analysis, and reporting complied with the Strengthening the Reporting of Observational Studies in Epidemiology (STROBE) guidelines and the Transparent Reporting of a Multivariable Prediction Model for Individual Prognosis or Diagnosis (TRIPOD) statement, respectively.^42^, ^43^

### Ethics

The prospective cohort study has been approved by the local ethics committee (Ethikkommission Nordwest- und Zentralschweiz EKNZ - https://www.eknz.ch) and was conducted in compliance with the declaration of Helsinki and its amendments.^44^ Informed consent was primarily obtained from patients directly. In patients without the capacity of judgment, informed consent was obtained from surrogate decision-makers according to Swiss legal regulations.

### Data collection and measures

Data was prospectively collected from the digital ICU patient-data management system and the medical records of the University Hospital Basel. The following data was collected for the purpose of this study:

‐ Baseline characteristics (Age, sex, and comorbidities)
‐ Cardiac arrest-related data (Cardiac arrest etiology, no-flow time [time from the beginning of cardiac arrest until the beginning of basic life support measures], low-flow time [time from the beginning of basic life support measures until ROSC], time until ROSC [no-flow time + low-flow time], the initial rhythm of cardiac arrest [i.e., shockable, non-shockable], cardiac arrest circumstances [observed/non-observed, public/private/in-hospital, professional/non-professional bystander cardiopulmonary resuscitation], epinephrine dosing during resuscitation)
‐ Laboratory values at hospital/ICU admission (e.g., pH, lactate levels, neuron-specific enolase, potassium, etc.) and on the following seven days or until ICU discharge (maximum seven days). For this study we used the laboratory values recorded ad ICU admission.
‐ Clinical parameters at hospital/ICU admission (Glasgow Coma Score [GCS], endotracheal intubation, haemodynamic support [mechanical/pharmacological].

### Post-cardiac arrest scores

The predictive performance of ChatGPT-4 was compared to three post-cardiac arrest scores that can be used to predict outcomes after cardiac arrest: The OHCA score, the Cardiac Arrest Hospital Prognosis (CAHP) score, and the PROLOGUE score (PROgnostication using LOGistic regression model for Unselected adult cardiac arrest patients in the Early stages). All three scoring systems have been repeatedly validated.^8^, ^30^, ^45^ The scores integrate different parameters that have been associated with outcomes after cardiac arrest: Personal, cardiac arrest-related, and clinical/laboratory parameters upon hospital and/or ICU admission. An overview of the individual scores can be obtained from the online-only supplement (**eTable 1**).^9^, ^10^, ^11^ For the calculation of the respective cardiac arrest scores, the methodology of the original publications was strictly followed.^9^, ^10^, ^11^

### Outcomes

The primary outcome was defined as in-hospital mortality. The secondary outcome was poor neurological outcome at hospital discharge measured by the Cerebral Performance Category (CPC), which is recommended by international expert consensus.^46^, ^47^ The CPC system classifies the neurological outcome after cardiac arrest into five different levels: CPC = 1: Good neurological recovery; CPC = 2: Moderate cerebral disability; CPC = 3: Severe cerebral disability; CPC = 4: Persistent vegetative state or coma; CPC = 5: Death including brain death.^48^ In accordance with expert consensus and previous research in the field, the neurological outcome was then dichotomized into good outcome (CPC 1–2) and poor outcome (CPC 3–5).^46^, ^47^

### Development of the chat prompt and data extraction from ChatGPT-4

For the development of a standardized chat prompt, we utilized an iterative approach as suggested by Kanjee et al.^17^ An introductory text was drafted and refined by trial and error until the desired responses were given by ChatGPT-4. The introductory text rigorously explained the task and the setting to the LLM. The complete standardized chat prompt can be obtained from the online-only supplement (**eMethods 1**). In brief, the LLM was asked to put itself into the position of an ‘AI intensive care doctor’ receiving a cardiac arrest patient with ROSC in his intensive care unit. Also, the LLM was provided with sixteen patient-related parameters. These have been selected as they are well-known predictors of outcomes after cardiac arrest and are all included in one or more of the post-cardiac arrest scores (OHCA, CAHP, PROLOGUE). Furthermore, uploading unstructured data in the form of medical charts to a cloud-based LLM would cause significant issues regarding data privacy. The following sixteen parameters were provided: Age, sex, observed cardiac arrest, setting, initial rhythm, no-flow time, low-flow time, epinephrine administration during resuscitation, pH at ICU admission, potassium level at ICU admission, lactate level at ICU admission, haemoglobin level at ICU admission, phosphate level at ICU admission, creatinine level at ICU admission, pupillary light reflex at ICU admission, GCS motor score at ICU admission. The LLM was then asked to provide replies to the following two questions:

‐ Will this patient survive to hospital discharge? Please provide a yes/no answer and the probability of survival in percent.
‐ Will this patient experience a good neurological outcome at hospital discharge as defined by a cerebral performance category scale of 1 or 2? Please provide a yes/no answer and the probability of a good neurological outcome in percent.

The chat prompt for each patient was generated by a pre-programmed Excel (Microsoft, Redmond, Washington, USA) spreadsheet (**eMethods 2**), which combined the standardized chat prompt with the cardiac arrest parameters of each patient, which allowed to copy-paste the whole chat prompt in a single command thereby reducing the possibility of erroneous data entries.

The LLM’s answers to the questions were then registered in a separate Excel (Microsoft, Redmond, Washington, USA) spreadsheet. We verified that the LLM would assess each patient individually by re-opening a new chat after each patient. In total, we performed three runs so that each patient was assessed three times by the LLM. Regarding the dichotomous yes/no answers, the most frequent answer of the three runs was counted, e.g., if the individual answers were yes/yes/no, the overall answer was registered as yes. Regarding the probability of survival and the probability of good neurological outcome in percent, the mean value of the three runs was used for statistical analysis. All chat prompts, including answers, have been thoroughly documented by screenshots. If the LLM provided non-logical answers (i.e., hallucinations), such as providing a higher probability of survival with a good neurological outcome than survival, the LLM was asked to reconsider its answer, also using a standardized text input. For the statistical analysis, the corrected, logical answers were used.

### Statistical analysis

To characterize the patient cohort, descriptive statistics, including means (±SD), were used for continuous variables, whereas frequencies were reported for binary or categorical variables. Receiver operating characteristics (ROC) and corresponding areas under the curve (AUC) were created to evaluate the prognostic performance of ChatGTP-4 to predict outcomes and to compare it to the OHCA, CAHP, and Prologue scores. We calculated sensitivity, specificity, positive and negative predictive values, and likelihood ratios for mortality and poor neurological outcome predicted by ChatGTP-4. Missing data was handled by multiple imputations based on chained equations to enhance the completeness of the dataset, mitigate biases arising from missing data, and contribute to more robust and reliable analyses, thus strengthening the validity of our study findings. Imputations were calculated using multiple covariables (i.e., socio-demographics, comorbidities, resuscitation information, vital signs), including main outcomes (death, neurological outcome) as suggested by Sterne et al.^49^ STATA 15.0 was used for statistical analyses, and a two-sided p-value of <0.05 was considered significant.

## Results

### Baseline characteristics

Of the 713 included patients, 309 patients died in hospital, and 387 had a poor neurological outcome (including CPC 5 = death) at hospital discharge. The baseline characteristics of the cohort overall and stratified based on survival status are shown in Table 1. Factors significantly associated with mortality were higher age, pre-existing comorbidities (e.g., diabetes, chronic obstructive pulmonary disease, malignant disease), cardiac arrest at home, unwitnessed arrest, non-shockable initial heart rhythm, longer time to ROSC, no bystander CPR, longer no-flow and low-flow time, higher doses of epinephrine during resuscitation, non-reactive pupils and a low Glasgow coma scale motor score at ICU admission.

**Table 1:** Baseline characteristics.

	n	All	Survivors to hospital discharge (n = 404)	In-hospital Death (n = 309)	p-value
Factors included in chat prompt					
Age, mean (SD)	713	64.8 (14.4)	62.9 (14.2)	67.4 (14.2)	<0.001
Female, n (%)	713	198 (27.8%)	98 (24.3%)	100 (32.4%)	0.018
*Cardiac arrest setting*	*703*				
At home		262 (37.3%)	116 (29.2%)	146 (47.7%)	<0.001
In public		322 (45.8%)	212 (53.4%)	110 (35.9%)	
In-hospital		119 (16.9%)	69 (17.4%)	50 (16.3%)	
Witnessed	712	578 (81.2%)	361 (89.4%)	217 (70.5%)	<0.001
*Initial rhythm of cardiac arrest*	*711*				
Non-shockable		341 (48.0%)	134 (33.2%)	207 (67.4%)	<0.001
Shockable		370 (52.0%)	270 (66.8%)	100 (32.6%)	
No-flow time, min, mean (SD)	583	3.02 (5.26)	1.63 (3.49)	5.02 (6.58)	<0.001
Low-flow time, min, mean (SD)	675	19.33 (17.12)	15.54 (13.85)	24.15 (19.53)	<0.001
*Epinephrine during resuscitation, n (%)*	*669*				
No epinephrine		251 (37.5%)	195 (51.7%)	56 (19.2%)	<0.001
>0 to < 3 mg epinephrine		208 (31.1%)	100 (26.5%)	108 (37.0%)	
>3mg epinephrine		210 (31.4%)	82 (21.8%)	128 (43.8%)	
*Levels of routine blood markers*					
pH, mean (SD)	626	7.21 (0.17)	7.25 (0.134)	7.15 (0.184)	<0.001
Potassium, mean (SD)	694	4.32 (0.81)	4.23 (0.75)	4.49 (0.88)	0.001
Lactate, mean (SD)	686	6.48 (4.43)	4.94 (3.4)	8.39 (4.81)	<0.001
Hemoglobin, g/l, mean (SD)	692	132 (24)	135 (22)	127 (25.7)	<0.001
Creatinine, µmol/l mean (SD)	678	113 (79.7)	105 (91.4)	123 (60.2)	0.006
Phosphate, mean (SD)	707	1.60 (0.76)	1.38 (0.56)	1.89 (0.86)	<0.001
*Pupil reaction at ICU admission*	*604*				
Not reactive		103 (16.1%)	14 (3.9%)	89 (32.0%)	<0.001
Reactive		537 (83.9%)	348 (96.1%)	189 (68.0%)	
Glasgow Coma Scale, motor score, at ICU admission mean (SD)	708	2.30 (1.99)	2.97 (2.20)	1.43 (1.22)	<0.001
Cardiac arrest characteristics					
Time to ROSC, min, mean (SD)	566	22.03 (18.31)	16.91 (13.59)	29.29 (21.44)	<0.001
Bystander CPR	712	507 (71.2%)	324 (80.2%)	183 (59.4%)	<0.001
*Reason for Cardiac arrest*	708				
Coronary heart disease, n (%)		335 (47.3%)	229 (57.4%)	106 (34.3%)	<0.001
Primary arrhythmia		103 (14.5%)	63 (15.8%)	40 (12.9%)	
Other/unclear		270 (38.1%)	107 (26.8%)	163 (52.8%)	
Comorbidities					
Coronary heart disease, n (%)	712	411 (57.7%)	252 (62.4%)	159 (51.6%)	0.005
Congestive heart failure, n (%)	711	101 (14.2%)	52 (12.9%)	49 (16.0%)	0.28
COPD, n (%)	712	78 (11.0%)	25 (6.2%)	53 (17.2%)	<0.001
Liver disease, n (%)	712	19 (2.7%)	9 (2.2%)	10 (3.2%)	0.48
Hypertension, n (%)	712	368 (51.7%)	214 (53.0%)	154 (50.0%)	0.45
Diabetes, n (%)	712	155 (21.8%)	75 (18.6%)	80 (26.0%)	0.022
Chronic kidney disease, n (%)	712	98 (13.8%)	53 (13.1%)	45 (14.6%)	0.58
Malignant disease, n (%)	711	79 (11.1%)	30 (7.4%)	49 (16.0%)	<0.001
Neurological disease, n (%)	712	103 (14.5%)	51 (12.6%)	52 (16.9%)	0.13

**Table 1.** Baseline characteristics of the study population stratified according to the primary outcome (in-hospital mortality).

Abbreviations: COPD Chronic obstructive pulmonary disease; CPR Cardiopulmonary resuscitation; ICU Intensive care unit; ROSC Return of Spontaneous Circulation; SD standard deviation.

### Mortality prediction by ChatGPT-4 compared with post-cardiac arrest scores

**Fig. 1:**
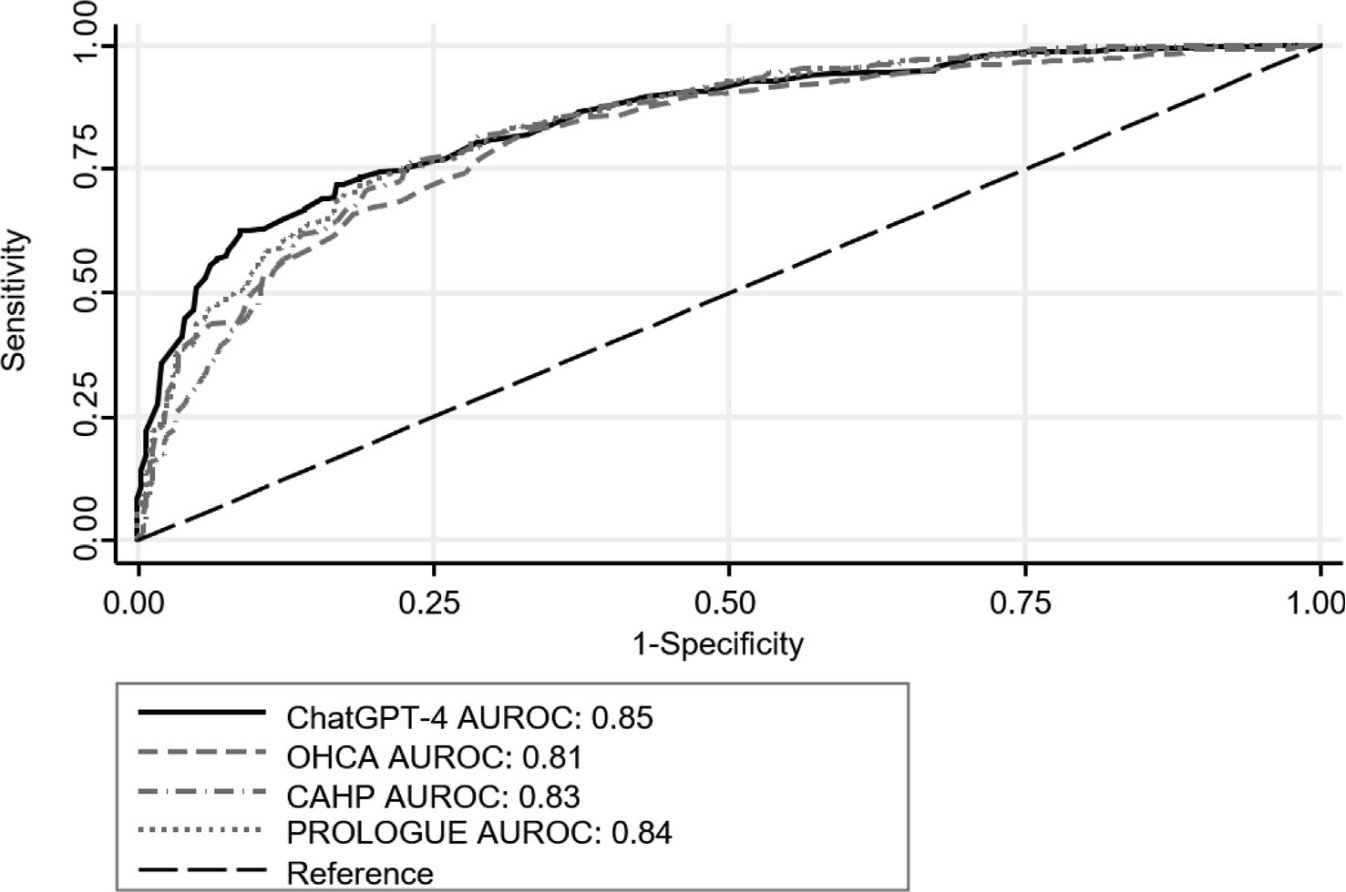
**Comparison of ROC curves for mortality at hospital discharge**. Abbreviations: AUROC Area under the receiver operating characteristics curve; CAHP Cardiac arrest hospital prognosis; ChatGPT-4 Chat Generative Pre-Trained Transformer 4; OHCA Out-of-hospital cardiac arrest; PROLOGUE Prognostication using logistic regression model for unselected adult cardiac arrest patients in the early stages.

Mortality at hospital discharge was 43% (95% CI 40% to 47%; n = 309). The mean predicted mortality by ChatGTP-4 was 44% (95% CI 42 to 46%). Overall, the AUROC of ChatGTP-4 was 0.85, similar to the predictive performance of the OHCA (AUROC 0.81), CAHP (AUROC 0.83), and Prologue (AUROC 0.84) scores (Fig. 1).

In addition to the probabilities, we also looked at the prediction of mortality as binary outcomes. ChatGTP-4 predicted death in 229 patients and survival in 484 patients. Overall, ChatGTP-4′s positive predictive value (PPV) was 85% (194/229), and the negative predictive value (NPV) was 76% (369/484), resulting in a sensitivity and specificity of 63% and 91%, respectively (Table 2).

**Table 2:** Prognostic measures of ChatGPT-4.

	In-hospital mortality	Poor Neurological Outcome
Prevalence %, (95%CI)	43.3 (39.7–47.1)	54.3 (50.5–58.0)
Sensitivity %, (95%CI)	62.8 (57.1–68.2)	87.9 (84.2–90.9)
Specificity %, (95%CI)	91.3 (88.2–93.9)	49.1 (43.5–54.6)
Positive likelihood ratio, (95%CI)	7.25 (5.2–10.1)	1.73 (1.5–1.9)
Negative likelihood ratio, (95%CI)	0.41 (0.4–0.5)	0.25 (0.2–0.3)
Odds ratio, (95%CI)	17.79 (11.7–26.9)	6.97 (4.8–10-1)
Positive predictive value %, (95%CI)	84.7 (79.4–89.1)	67.2 (62.9–71.3)
Negative predictive value %, (95%CI)	76.2 (72.2–80.0)	77.3 (71.0–82.8)

**Table 2.** Performance of ChatGPT-4 for the prediction of in-hospital mortality and poor neurological at hospital discharge (Cerebral Performance Category Scale 3–5 including death).

Abbreviations: ChatGPT-4 Chat Generative Pre-Trained Transformer 4*,* CI confidence interval.

### Prediction of poor neurological outcome by ChatGPT-4 compared with post-cardiac arrest scores

**Fig. 2:**
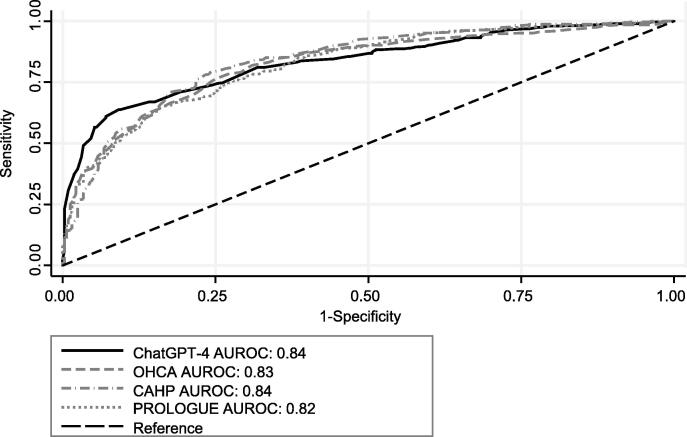
**Comparison of ROC curves for poor neurological outcome at hospital discharge (Cerebral Performance Category Scale 3**–**5 including death).** Abbreviations: AUROC Area under the receiver operating characteristics curve; CAHP Cardiac arrest hospital prognosis; ChatGPT-4 Chat Generative Pre-Trained Transformer 4; OHCA Out-of-hospital cardiac arrest; PROLOGUE Prognostication using logistic regression model for unselected adult cardiac arrest patients in the early stages.

Poor neurological outcome at hospital discharge was 54% (95% CI 51% to 58%; n = 387). The mean predicted probability of the ChatGTP-4 was 61% (95% CI 60% to 63%). Overall, the AUROC of ChatGTP-4 for poor neurological outcome was 0.84, which was again similar to the OHCA (AUROC 0.83), CAHP (AUROC 0.84), and Prologue (AUROC 0.82) scores (Fig. 2).

ChatGTP-4 predicted a poor neurologic outcome in 506 patients and a good neurological outcome in 207 patients. Overall, the PPV was 67% (340/506), and the NPV was 77% (160/207), resulting in a sensitivity and specificity of 88% and 49%, respectively (Table 2).

## Hallucinations of ChatGPT-4 concerning the prediction of probabilities

In all three runs of the ChatGPT-4 experiment, instances of hallucinations occurred in the form of irrational responses to the input prompts provided to ChatGPT-4. Specifically, we observed irrational responses in 59 out of 713 cases (8.3%), 94 out of 713 cases (13.2%), and 100 out of 713 cases (14.0%) in the first, second, and third run, respectively. When directly entering a standardized prompt requesting a correction, all illogical responses were subsequently replaced with logical and coherent answers. The prognostic performance of the uncorrected prediction, however, was similar to the final results regarding mortality (AUROC of 0.84) and inferior regarding neurological outcome (AUROC of 0.75).

## Discussion

This study compared the prognostic value of a large language model (ChatGPT-4) for prognostication in cardiac arrest patients with that of well-validated and established cardiac arrest scores. The prognostic performance of ChatGPT-4 for predicting mortality and poor neurological outcomes was good and in the range of the validated post-cardiac arrest scores, demonstrating the potential capabilities of artificial intelligence in clinical practice. However, some findings need further discussion.

First, in about 14% (300/2139) of chat queries, the untrained ChatGPT-4 generated illogical answers (i.e., hallucinations), such as a higher probability of poor neurological outcome compared to the probability of death. Here, we asked ChatGPT-4 to reconsider and correct the prediction, which was done without generating further illogical answers. This illustrates that artificial intelligence still may be used most efficiently when combined with ‘human intelligence’, i.e., an experienced clinician. Furthermore, this emphasizes that the use of LLMs in clinical practice needs close supervision by its user.

End-of-life decisions are inherently difficult and require a high level of exclusively human qualities such as professional experience, compassion, emotions, and consciousness of cultural backgrounds and social inequalities. However, LLMs are solely machines that base decisions on stochastic principles without consciousness or emotions.

Although there is an increasing number of studies using LLMs in medicine, studies assessing LLM’s prediction skills for patient outcomes are scarce. In a small study including 30 emergency department patients, ChatGPT-3.5 and -4′s ability to generate a meaningful differential diagnosis was comparable to medical experts. However, a potential association with outcomes was not assessed.^19^ In a preprint online publication investigating the performance of three large LLMs (ChatGPT-3.5, ChatGPT-4, Bard) for the prediction of 10-year cardiovascular risk, the LLM’s performance was comparable to the Framingham score.^23^

Although the performance of LLM in predicting medical outcomes seems promising, important limitations need to be addressed. First, the predictive value does not significantly exceed known validated post-cardiac arrest scores. As the positive predictive value for mortality and/or poor neurological outcome are not satisfactory, clinicians should never base their decisions regarding withdrawal of life-sustaining therapies on single tests or scores. This is reflected in the clinical guidelines recommending a multimodal approach without the use of post-cardiac arrest scores.

Also, clinicians assessing LLMs should be aware of the ‘stochastic parrot’ principle proposed by Bender et al.^50^ and emphasized by Boussen et al.^51^. Due to the underlying algorithm, an LLM does neither understand the input that is entered nor the output generated. It just rigidly repeats structures and patterns it has been trained on, including prejudices, stereotypes, and social inequalities.^52^ This might be an explanation for the comparable but not significantly better performance of ChatGPT-4 in the prediction of mortality and neurological outcomes when compared to validated post-cardiac arrest scores. This aligns with other studies finding comparable, but not superior, performances in clinical or theoretical contexts.^17^, ^53^ Due to the algorithm behind LLMs, the user should be aware of a certain number of ‘hallucinations’ or illogical answers generated. Hallucinations are a well-known shortcoming of LLMs and are associated with the stochastic parrot principle.^50^, ^51^ However, in our study, the rate of hallucinations was considerably low, with a maximum value of 14.1% per run. Nevertheless, ChatGPT-4′s ability to detect illogical answers is limited and still warrants the presence of a human controller.^50^, ^51^, ^54^

Additionally, ChatGPT-4 provided inconsistent answers in some patients, which we tried to account for by using the most frequent answer out of the three runs. However, this is a major limitation the use of ChatGPT-4 in prognosticating outcomes after cardiac arrest.

The field of LLM in medicine is exponentially increasing, as will the capabilities of LLMs. Hence, future research should focus on the evaluation of performance-enhancing plugins, which might have the ability to reduce the production of false results and/or references by checking the results with external databases such as PubMed.^55^ Furthermore, specific training of healthcare professionals and transforming medical datasets into easily accessible and structured databases will be crucial to improving the value of LLMs for clinical questions, as recently shown in a study integrating an LLM in the clinical workflow.^28^ Also, further specific training of the LLM is warranted to enable the LLM to perform significantly better than validated scores. However, specific training requires a training dataset which can be difficult to obtain, if considering patient data safety. Training an LLM with unstructured medical charts might involuntarily expose patients’ identities or upload confidential data to a cloud-based LLM. In the present study this issue was addressed through uploading anonymized and structured patient data. Furthermore, training data must be well chosen and representational for the training purpose, as otherwise real-world bias might be reproduced by the LLM.

At the moment of prompting ChatGPT-4 was designed to answer queries based on its training data only, and its current knowledge did not extend beyond September 2021. Furthermore, the ‘black box problem’, describing the current lack of understanding of the underlying algorithm and its method of solving, remains an issue. This is in line with the recently published expert opinion,^56^ that we need to ensure that these models are safe and effective through vigorous testing, uncovering possible biases, and thereby enabling a correction and training of the models.^54^

Future research should focus on the direct integration of LLMs into clinical information systems, which could substantially decrease the administrative workload for physicians, allowing a focus on patient care as a historic core competence. However, concerns regarding data privacy will be significant.

### Strengths and limitations

To the best of our knowledge, this is the first study assessing the prognostication of outcomes after cardiac arrest by an LLM using real-world data. A pragmatic approach aiming at high reproducibility and data integrity using an established post-cardiac arrest database was used. However, the present study also has several limitations.

First, the parameters used for prognostication were also available to the clinicians involved in WLST. Hence, there might be a certain risk of self-fulfilling prophecies.^57^, ^58^ In addition, the studies the LLM has been exposed to might also have been influenced by self-fulfilling prophecies. Hence, one cannot be sure to what extent the LLM can predict true outcomes or just reproduces the self-fulfilling prophecies present in studies the LLM has been exposed to.

Second, due to the algorithm behind LLMs, the user should be aware of a certain number of hallucinations or illogical answers generated.

Third, as ChatGPT-4 was not designed for healthcare purposes, its applicability and validity to answer specific clinical questions remains unclear and warrants further research. Fourth, our study is based on a single-center cohort, limiting its generalizability to other centers or regions and emphasizing the importance of future research in diverse contexts to enhance the external validity of the results.

## Conclusions

ChatGPT-4 showed a good performance in predicting mortality and poor neurological outcome comparable to validated post-cardiac arrest scores and thus may be a helpful future tool for early risk prediction in adult cardiac arrest patients. However, due to frequent hallucinations in the output data, ChatGPT-4 still needs human supervision. Also, training a specific future LLM needs structured medical data sets, and future research should focus on validation of LLMs in various clinical settings.

## Funding

Sabina Hunziker and her research team were supported by the Swiss National Foundation (SNF) (Ref 10001C_192850/1 and 10531C_182422) and the Gottfried Julia Bangerter-Rhyner Foundation (8472/HEG-DSV) and the Swiss Society of General Internal Medicine (SSGIM).

## CRediT authorship contribution statement

**Simon A. Amacher:** Writing – review & editing, Writing – original draft, Visualization, Project administration, Methodology, Formal analysis, Data curation, Conceptualization. **Armon Arpagaus:** Writing – review & editing, Writing – original draft, Visualization, Project administration, Methodology, Formal analysis, Data curation, Conceptualization. **Christian Sahmer:** Writing – review & editing, Data curation. **Christoph Becker:** Writing – review & editing. **Sebastian Gross:** Writing – review & editing. **Tabita Urben:** Writing – review & editing. **Kai Tisljar:** Writing – review & editing. **Raoul Sutter:** Writing – review & editing. **Stephan Marsch:** Writing – review & editing. **Sabina Hunziker:** Writing – review & editing, Writing – original draft, Visualization, Methodology, Investigation, Data curation, Conceptualization.

## Declaration of competing interest

The authors declare that they have no known competing financial interests or personal relationships that could have appeared to influence the work reported in this paper.
