# Supplementary material for: Prediction of outcomes after cardiac arrest by a generative artificial intelligence model
Source: Resusc Plus. 2024 Feb 22;18:100587. doi: 10.1016/j.resplu.2024.100587 (PMC10906512; doi:10.1016/j.resplu.2024.100587)
Supplement: Supplementary data 1 [file mmc1.docx]

**Appendix**

**Appendix A Chat prompts**

**Appendix B Excel code for generating the standardized chat prompt**

**Appendix C Table C1 Overview of post-cardiac arrest scores**

**Appendix A Chat prompts**

**Standardized introductory text to the chat prompt**

*I am running an experiment on outcome prediction in patients after cardiac arrest. This experiment will include patients after in-hospital or out-of-hospital cardiac arrest.*

*You are not going to treat any patients, and your decisions will have no influence on any real patients.*

*I will provide you with 16 parameters that have been proven in the past to be important prognostic factors in patients with cardiac arrest. Imagine being an artificial intelligence intensive care unit doctor who receives the patient after the return of spontaneous circulation (ROSC) in the intensive care unit. After checking the 16 parameters of the medical case, I want you to answer the following two questions.*

*Please provide only a yes/no answer, and do not explain your reasoning.*

*1)Will this patient survive to hospital discharge? Please provide a yes/no answer.*

*2)**Will this patient experience a good neurological outcome at hospital discharge as defined by a cerebral performance category scale of 1 or 2. Please provide a yes/no answer.*

**Appendix B Excel code for generating the standardized chat prompt**

**Design of a basic data table generating a text prompt**

1. Step 1:16 parameters (Age, sex, observed cardiac arrest, setting, initial rhythm, no-flow time, low-flow time, epinephrine administration during resuscitation, pH at ICU admission, potassium level at ICU admission, lactate level at ICU admission, haemoglobin level at ICU admission, phosphate level at ICU admission, creatinine level at ICU admission, pupillary light reflex at ICU admission, GCS motor score at ICU admission) included in the chat prompt were extracted from the COMMUNICATE/PROPHETIC-cohort database for all patients. Data was checked for consistency and rounded to two decimal places, where necessary.
2. Step 2: For each column of a parameter, a new column was generated. A description of the data and the data itself was inserted using the command CONCATENATE().
   i.e. =CONCATENATE("Age: ";ROUND(B8;0); " years")
3. Step 3: The complete data of the 16 columns, including the parameters and their description, were then merged with an introductory text to the column, representing the chat prompt copied into the web browser.
   i.e. = CONCATENATE("Introductory text:"; CHAR (10);"Baseline characteristics:"; CHAR (10);R2; CHAR (10);S2; CHAR (10); CHAR (10);"Cardiac Arrest Data:"; CHAR (10);U2; CHAR (10);T2; CHAR (10);V2; CHAR (10);W2; CHAR (10);X2; CHAR (10);Y2; CHAR (10); CHAR (10);"Laboratory data:"; CHAR (10);Z2; CHAR (10);AA2; CHAR (10);AB2; CHAR (10);AC2; CHAR (10);AD2; CHAR (10);AE2; CHAR (10); CHAR (10);"Clinical examination:"; CHAR (10);AF2; CHAR (10);AG2)

| Table C1 Overview of post-cardiac arrest scores | | |
| --- | --- | --- |
| OHCA | **CAHP** | **PROLOGUE** |
| Initial rhythm: VF or VT [yes/no] | Age [years] | Unwitnessed collapse |
| No-flow interval [min] | Arrest setting [home/public] | Potassium ≥ 4.4 mEq/L |
| Low-flow interval [min] | Shockable rhythm [yes/no] | Lactate ≥ 8 mmol/l |
| Serum creatinine [µmol/L] | No-flow interval [min] | Adrenaline dose ≥ 2 mg |
| Arterial lactate [mmol/L] | Low-flow interval [min] | Low-flow duration ≥ 18 min |
|  | pH at admission | Hemoglobin < 13.2 g/dl |
|  | Dosage of epinephrine administered [0, 1–2 or ≥ 3 mg] | Creatinine ≥ 1.21 mg/dl |
|  |  | Phosphate ≥ 5.8 mg/dl |
|  |  | Non-shockable rhythm |
|  |  | Absent pupillary light reflex |
|  |  | Age ≥ 59 years |
|  |  | Glasgow Coma Scale motor score < 2 |
| eTable 1. Overview of cardiac arrest scores including parameters. All parameters are either assessed at hospital or intensive care unit admission.  Abbreviations: OHCA Out of Hospital Cardiac arrest Score, CAHP Cardiac Arrest Hospital Prognosis Score, PROLOGUE PROgnostication using LOGistic regression model for Unselected adult cardiac arrest patients in the Early stages Score. | | |

**Appendix C Overview of post-cardiac arrest scores**
